# Prognostic value of combined stratification using TyG index and CD4^+^ T cell count for 28-day all-cause mortality risk in patients with HIV infection and sepsis: a retrospective cohort study

**DOI:** 10.3389/fmed.2025.1688334

**Published:** 2026-01-12

**Authors:** Yongchang Wu, Jiejing Chen, Yu Meng, Mingyue Ren, Zhenyi Zou, Linghua Li, Xilong Deng, Yueping Li

**Affiliations:** 1 Institute of Infectious Diseases, Guangzhou Eighth People’s Hospital, Guangzhou Medical University, Guangzhou, Guangdong, China; 2 Infectious Disease Center, Guangzhou Eighth People’s Hospital, Guangzhou Medical University, Guangzhou, Guangdong, China; 3 Department of Critical Care Medicine, Guangzhou Eighth People’s Hospital, Guangzhou Medical University, Guangzhou, Guangdong, China

**Keywords:** HIV, sepsis, TyG, CD4, insulin resistance

## Abstract

**Background:**

Sepsis contributes to global mortality due to chronic immunodeficiency and metabolic disturbances, particularly in those infected with the human immunodeficiency virus (HIV). The TyG index and CD4^+^ T-cell count individually predict sepsis outcomes, but reflect different biological aspects. The benefit of integrating these markers for risk stratification in patients with HIV-associated sepsis remains unclear. This study aimed to assess whether combining the TyG index, a surrogate for insulin resistance, with CD4^+^ T cell count enhanced early prediction of 28-day mortality risk in adults hospitalized with HIV-associated sepsis.

**Methods:**

Clinical data were retrospectively collected from patients admitted to the Eighth Affiliated Hospital of Guangzhou Medical University. The participants were stratified into four risk categories according to their TyG index and CD4^+^ T cell count. The primary outcome was 28-day all-cause mortality, and secondary outcomes were 7-day and in-hospital all-cause mortality. Survival across strata was compared using the Kaplan–Meier analysis and the log-rank test; associations were determined using multivariate Cox proportional hazards models. Independent predictors of death were ranked using the Boruta feature selection algorithm. In a nested sub-cohort of 155 patients, plasma concentrations of 13 inflammatory cytokines were quantified using the LEGENDplex Human Inflammation Panel 1.

**Results:**

Among 1,278 patients with HIV-associated sepsis, 847 had CD4 counts <50 cells/μL. The Kaplan–Meier analysis revealed progressively higher 7-day and 28-day all-cause mortality rates across these strata, with the highest mortality in the low CD4^+^ and high TyG groups. A multivariable Cox regression analysis, adjusted for multiple covariates, indicated an elevated risk of 28-day mortality in patients with low CD4 and high TyG compared to those with high CD4 and low TyG. Similar trends were observed for 7-day and in-hospital mortality. The Boruta algorithm identified combined TyG-CD4 as an important predictor. The immunological sub-study demonstrated significant differences in cytokine profiles among the risk groups, with the low CD4 and high TyG groups exhibiting a specific inflammatory response.

**Conclusion:**

A combined TyG-CD4 assessment provides a rapid and straightforward means of integrating metabolic and immunological insights to identify high-risk individuals early, thereby generating hypotheses for future trials of personalized therapeutic strategies.

## Graphical abstract

**Figure:**
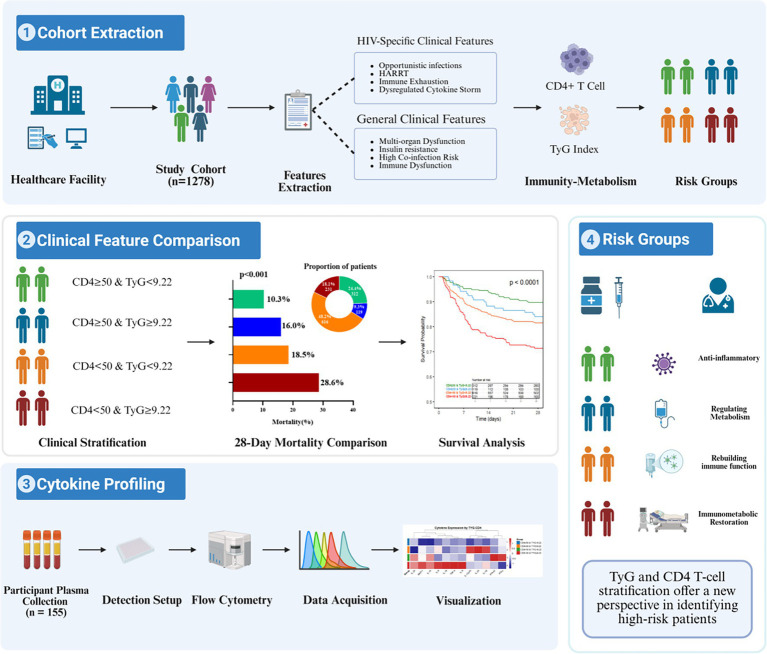
Figure created with BioRender.com.

## Background

Sepsis is a life-threatening syndrome characterized by organ dysfunction caused by a dysregulated host response to infection. More than 10 million deaths occur annually—constituting approximately 20% of all global deaths—due to sepsis (1, 2). Profound metabolic derangements that accompany sepsis include insulin resistance (IR), disordered glucose handling, altered lipid metabolism, amplified systemic inflammation, and worse clinical outcomes (3–5).

People living with HIV (PLWH) are at a particularly high risk of developing sepsis and fatal consequences owing to the intersecting effects of chronic immune activation, immunodeficiency, and metabolic dysfunction (6, 7). Their complex pathophysiological state requires more nuanced prognostic tools. The TyG index, a simple and cost-effective surrogate marker of IR and broader metabolic impairment, has been independently validated as a predictor of adverse outcomes in sepsis (8). For instance, elevated TyG values are associated with higher in-hospital mortality among patients with sepsis, underscoring the relevance of this index during acute inflammatory states (9). Circulating CD4^+^ T-cell count is the gold-standard measure of immune injury and opportunistic infection risk in HIV infection and is tightly coupled with patient prognosis (10). Low CD4^+^ counts in PLWH frequently coexist with metabolic disturbances. Emerging evidence indicates that CD4 depletion may exacerbate IR via intensified inflammation and metabolic dysregulation, thereby increasing cardiovascular risk; this “metabolic–immune axis” likely contributes to poor outcomes in patients with HIV (11–13). Although CD4 count and TyG index individually capture critical aspects of immune and metabolic status, little is known about their combined value in predicting short-term mortality among PLWH with sepsis. A composite assessment can overcome the limitations of each marker alone, allowing for more precise risk stratification.

Against this backdrop, the purpose of this study was to assess whether the TyG index and CD4^+^ T-cell count at the onset of sepsis independently predict 28-day all-cause mortality in people living with HIV. We also profiled immune-metabolic signatures across risk strata to facilitate the earlier identification of high-risk patients and generate hypotheses for future targeted interventions.

## Methods

### Study design and data source

This retrospective cohort study used data from the electronic health record (EHR) system of Guangzhou Eighth People’s Hospital, a 2,000-bed tertiary referral center. Patients hospitalized between 1 January 2014 and 31 December 2024 were screened. Eligible patients satisfied the sepsis-3 definition of sepsis and had documented HIV infection confirmed by antibody or RNA testing (2).

Additionally, 155 participants were included for the comparison of serum cytokine levels, and no significant differences were observed in their baseline characteristics or comorbidities. Written informed consent was obtained from each participant before enrollment in the study, and the hospital’s ethics committee approved this study (Guangzhou Eighth People’s Hospital, Approval Number: 202033166).

**Figure 1:**
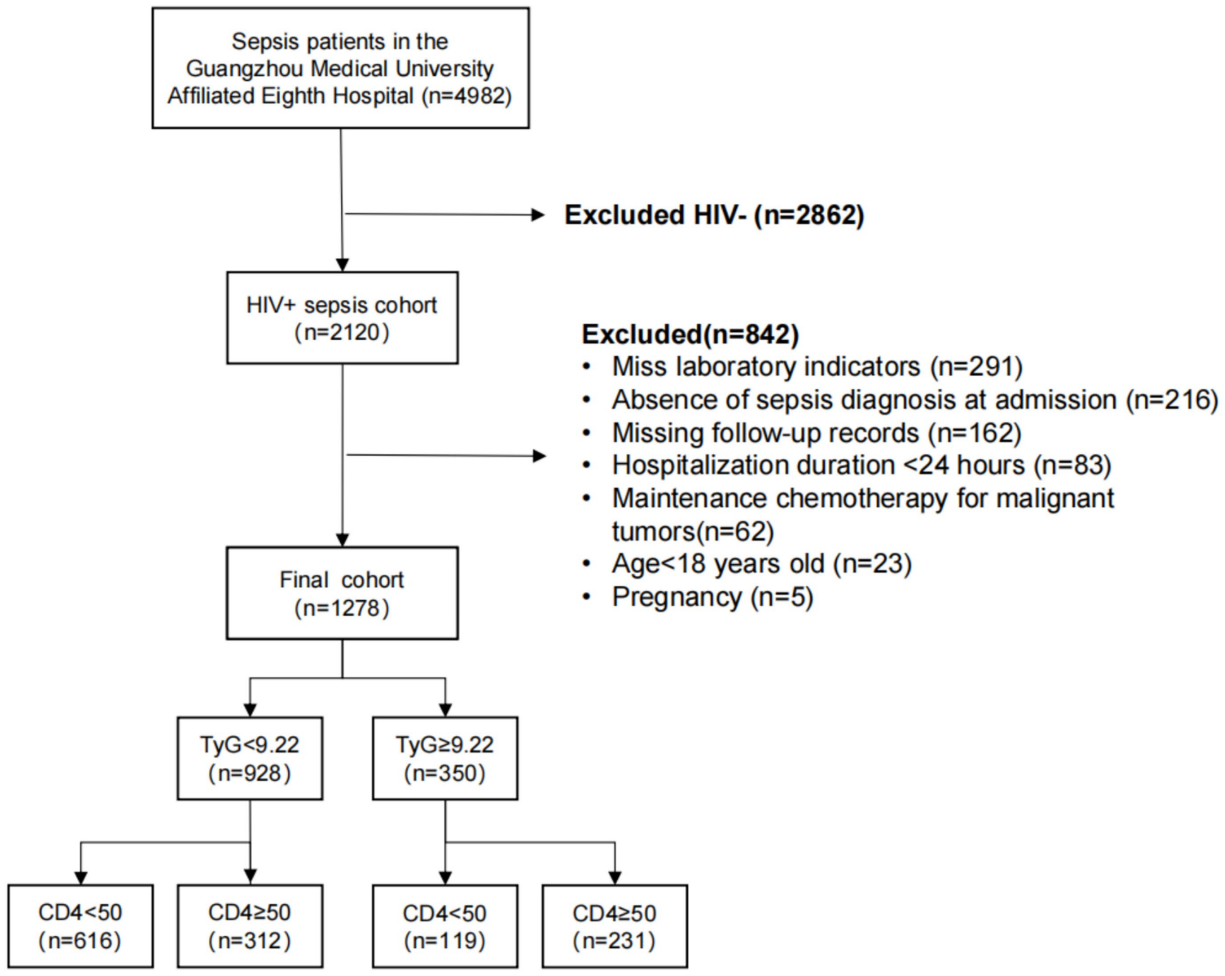
Flowchart of the study participants.

Consecutive adults with laboratory-confirmed HIV infection who met the sepsis-3 criteria upon admission were screened. The exclusion criteria were as follows: (1) missing key laboratory data, (2) age < 18 years, (3) hospital stay < 24 h, (4) ongoing chemotherapy for malignancy, (5) pregnancy, (6) an admission diagnosis other than sepsis, and (7) loss up to 28-day follow-up (Figure 1). Baseline laboratory tests were obtained within 48 h of sepsis onset (SOFA ≥ 2); the triglyceride-glucose (TyG) index was calculated as ln[fasting triglyceride (mg/dL) × fasting glucose (mg/dL)/2]. Among 1,278 eligible patients, four prognostic strata were created by combining the optimal TyG cut-off for 28-day mortality (9.22, determined using Youden’s index) with the guideline CD4^+^ Threshold for profound immunosuppression (50 cells/μL) (10). Vital status at 28 days was ascertained through telephone interviews and a review of readmission records.

### Data collection

Trained clinicians extracted the data, including age, sex, length of stay, SOFA, septic shock, ICU admission, and therapeutic interventions (antiretroviral therapy, vasopressor use, nephrotoxic agents, corticosteroids, continuous renal replacement therapy, and invasive mechanical ventilation) into a standardized electronic form. Laboratory variables included serum albumin, CD4^+^ T cell count, creatinine, urea, total bilirubin, lactate dehydrogenase, serum glucose, serum triglycerides, platelets, lymphocytes, white blood cells, and monocytes. The recorded comorbidities were chronic kidney disease, diabetes, hypertension, liver cirrhosis, chronic obstructive pulmonary disease, and opportunistic infections (*Talaromyces marneffei*, *Pneumocystis jirovecii* pneumonia, cryptococcosis, and candidiasis).

### Outcome

The primary outcome was 28-day all-cause mortality, and the secondary outcomes were 7-day all-cause and in-hospital mortality.

### Cytokine measurement

Plasma samples obtained after sepsis onset from 155 patients were stored at −80 °C until analysis. Levels of 13 cytokines (IL-1β, IFN-α2, IFN-γ, TNF-α, MCP-1, IL-6, IL-8, IL-10, IL-12p70, IL-17A, IL-18, IL-23, and IL-33) were quantified with the LEGENDplex^™^ Human Inflammation Panel 1 (BioLegend 740809) following the manufacturer’s protocol. Thawed plasma was incubated with capture beads and fluorescence-labeled antibodies at room temperature. A minimum of 300 beads per sample was acquired, and analyte concentrations were calculated from standard curves using the cloud-based LEGENDplex data-analysis suite.^1^

### Statistical analysis

All analyses were performed in R 4.4.0; a two-sided *p*-value of <0.05 denoted statistical significance. Continuous variables are presented as mean ± SD or median (IQR) and were compared using Student’s *t*-test or the Mann–Whitney *U*-test, while categorical variables are denoted as number (%) and were compared using *χ*^2^ or Fisher exact tests. Survival was evaluated using the Kaplan–Meier curves, log-rank tests, and Cox proportional hazards models, with proportional hazard assumptions verified by Schoenfeld residuals. Pre-specified subgroups were examined using interaction terms in the adjusted Cox models. Predictor importance was assessed using the Boruta algorithm, and the discrimination of TyG, CD4, and combined models was quantified using area under the curve (AUC) values and compared with DeLong tests. Laboratory variables with <20% missingness were multiply imputed (five chained-equation iterations); candidate covariates with a variance-inflation factor of ≥2.0 were excluded. The TyG cut-point for 28-day mortality was selected by maximizing the Youden index on the ROC curve.

## Results

### Baseline characteristics

Among the 1,278 patients with HIV + sepsis (Table 1), the mean (SD) age was 43.8 (11.7), and 84.0% (*n* = 1,074) of them were men. The median (IQR) CD4^+^ T cell count was 24.0 (8.0–82.8) cells/μL, the median TyG index was 8.9 (8.5–9.3), and the mean (SD) SOFA score was 5.3 (2.6). Septic shock occurred in 36.1, 15.5% were admitted to the ICU, and the median length of stay was 26.5 days.

**Table 1:** Baseline characteristics of the study population according to the TyG index and CD4^+^ T cell count.

Variable	Total (*n* = 1,278)	CD4 ≥ 50 & TYG < 9.22 (*n* = 312)	CD4 ≥ 50 & TYG ≥ 9.22 (*n* = 119)	CD4 < 50 & TYG < 9.22 (*n* = 616)	CD4 < 50 & TYG ≥ 9.22 (*n* = 231)	*p*-value
Age, mean ± SD, years	43.8 ± 13.9	48.6 ± 14.8	47.2 ± 13.3	41.5 ± 13.5	42.1 ± 12.1	<0.001
Male, *n* (%)	1,074 (84.0)	251 (80.4)	101 (84.9)	531 (86.2)	191 (82.7)	0.137
SOFA score, mean ± SD	5.3 ± 3.0	4.9 ± 2.8	5.6 ± 2.9	5.1 ± 2.9	6.1 ± 3.4	<0.001
Hospital length of stay, mean ± SD, days	26.5 ± 21.6	28.0 ± 25.0	22.7 ± 17.2	27.4 ± 20.7	24.3 ± 20.9	0.035
7-day mortality, *n* (%)	116 (9.1)	15 (4.8)	7 (5.9)	59 (9.6)	35 (15.2)	<0.001
28-day mortality, *n* (%)	231 (18.1)	32 (10.3)	19 (16.0)	114 (18.5)	66 (28.6)	<0.001
In-hospital mortality, *n* (%)	335 (26.2)	58 (18.6)	25 (21.0)	168 (27.3)	84 (36.4)	<0.001
ICU admission, *n* (%)	198 (15.5)	28 (9.0)	25 (21.0)	95 (15.4)	50 (21.6)	<0.001
Septic shock, *n* (%)	461 (36.1)	93 (29.8)	41 (34.5)	231 (37.5)	96 (41.6)	0.029
Comorbidities						
Chronic kidney disease, *n* (%)	54 (4.2)	22 (7.1)	12 (10.1)	13 (2.1)	7 (3.0)	<0.001
Liver cirrhosis, *n* (%)	68 (5.3)	30 (9.6)	9 (7.6)	23 (3.7)	6 (2.6)	<0.001
COPD, *n* (%)	79 (6.2)	24 (7.7)	10 (8.4)	35 (5.7)	10 (4.3)	0.276
Diabetes, *n* (%)	82 (6.4)	20 (6.4)	21 (17.6)	15 (2.4)	26 (11.3)	<0.001
Hypertension, *n* (%)	87 (6.8)	42 (13.5)	17 (14.3)	13 (2.1)	15 (6.5)	<0.001
Coronary heart disease, *n* (%)	49 (3.8)	21 (6.7)	6 (5.0)	14 (2.3)	8 (3.5)	0.006
Opportunistic infections						
Talaromycosis, *n* (%)	468 (36.6)	35 (11.2)	15 (12.6)	299 (48.5)	119 (51.5)	<0.001
*Pneumocystis pneumonia*, *n* (%)	219 (17.1)	23 (7.4)	6 (5.0)	144 (23.4)	46 (19.9)	<0.001
Cryptococcosis, *n* (%)	92 (7.2)	18 (5.8)	5 (4.2)	50 (8.1)	19 (8.2)	0.296
Candidiasis, *n* (%)	410 (32.1)	66 (21.2)	22 (18.5)	229 (37.2)	93 (40.3)	<0.001
Treatments						
Antiretroviral therapy, *n* (%)	433 (33.9)	151 (48.4)	65 (54.6)	148 (24.0)	69 (29.9)	<0.001
Heparin use, *n* (%)	91 (7.1)	27 (8.7)	14 (11.8)	29 (4.7)	21 (9.1)	0.008
Vasopressor use, *n* (%)	445 (34.8)	78 (25.0)	36 (30.3)	232 (37.7)	99 (42.9)	<0.001
Glucocorticoid use, *n* (%)	206 (16.1)	30 (9.6)	17 (14.3)	106 (17.2)	53 (22.9)	<0.001
Invasive mechanical ventilation, *n* (%)	227 (17.8)	31 (9.9)	22 (18.5)	116 (18.8)	58 (25.1)	<0.001
CRRT use, *n* (%)	92 (7.2)	15 (4.8)	12 (10.1)	37 (6.0)	28 (12.1)	0.003
Laboratory values						
CD4 count, median (IQR), cells/μL	24.0 (8.0–82.8)	139.5 (82.0–260.2)	141.0 (79.0–279.5)	11.0 (5.0–24.0)	12.0 (6.0–25.0)	<0.001
Glucose, median (IQR), mg/dL	102.6 (86.6–126.0)	98.6 (84.4–116.8)	133.2 (106.7–168.6)	96.7 (81.7–113.4)	127.3 (98.3–171.7)	<0.001
Triglycerides, median (IQR), mg/dL	136.4 (99.2–191.3)	108.1 (83.9–139.3)	236.5 (180.2–326.8)	123.1 (93.9–156.1)	227.6 (176.3–294.1)	<0.001
TyG index, median (IQR)	8.9 (8.5–9.3)	8.6 (8.3–8.9)	9.7 (9.4–10.0)	8.7 (8.4–9.0)	9.5 (9.3–9.8)	<0.001
Total bilirubin, median (IQR), μmol/L	10.5 (6.9, 19.4)	9.1 (6.4, 15.7)	11.5 (6.5, 20.0)	10.2 (6.9, 17.0)	14.2 (8.5, 28.9)	<0.001
Creatinine, median (IQR), μmol/L	73.0 (58.5, 99.4)	75.0 (60.0, 103.1)	94.0 (73.9, 147.2)	67.2 (55.0, 84.1)	78.0 (61.0, 128.4)	<0.001
Platelets, median (IQR), ×10^3^/μL	136.5 (63.0–225.0)	180.0 (102.8–272.0)	138.0 (78.5–230.0)	130.0 (56.0–219.2)	91.0 (44.0–164.5)	<0.001
Lymphocytes, median (IQR), ×10^9^/L	0.5 (0.3, 1.0)	1.0 (0.6, 1.4)	0.8 (0.6, 1.4)	0.4 (0.2, 0.6)	0.3 (0.2, 0.7)	<0.001
White blood cells, median (IQR), ×10^9^/L	5.1 (3.2, 8.1)	6.2 (4.1, 9.8)	6.8 (4.0, 10.7)	4.5 (2.9, 7.1)	4.6 (2.9, 7.2)	<0.001
Urea, median (IQR), mmol/L	5.1 (3.6, 8.6)	5.2 (3.7, 7.8)	7.8 (4.5, 13.6)	4.7 (3.4, 7.1)	6.5 (3.9, 12.2)	<0.001
Monocytes, median (IQR), ×10^9^/L	0.2 (0.1, 0.4)	0.4 (0.2, 0.6)	0.4 (0.2, 0.6)	0.2 (0.1, 0.3)	0.1 (0.1, 0.3)	<0.001
Lactate dehydrogenase, median (IQR), U/L	369.5 (255.0–651.2)	287.0 (220.5–382.0)	353.0 (242.5–614.5)	396.5 (268.0–659.0)	538.0 (340.0–1043.0)	<0.001
Albumin, median (IQR), g/L	27.0 (22.2, 32.2)	30.9 (26.0, 35.3)	29.3 (25.0, 34.9)	25.9 (21.0, 30.4)	25.2 (21.0, 30.0)	<0.001

TyG index, triglyceride-glucose index; SOFA, Sequential Organ Failure Assessment score; ICU, intensive care unit; COPD, chronic obstructive pulmonary disease; CRRT, continuous renal replacement therapy.

High TyG status was found to be associated with higher urea and creatinine levels and a greater prevalence of diabetes and coronary heart disease (Supplementary Table 4). Patients in the low CD4 count group showed more opportunistic infections, organ injury markers, and therapeutic interventions (Supplementary Table 5). The combined high-risk category (CD4 < 50 cells/μL and TyG ≥ 9.22) displayed the most severe phenotype, with lower platelet, monocyte, and albumin levels; higher LDH, CRP, and bilirubin concentrations; a mean SOFA score of 6.1; and the highest incidence of septic shock (41.6%). No multicollinearity was detected among the variables (all variance inflation factors < 2.0; Supplementary Table 2); the patterns of missing data are detailed in Supplementary Table 3.

### Primary outcome

**Figure 2:**
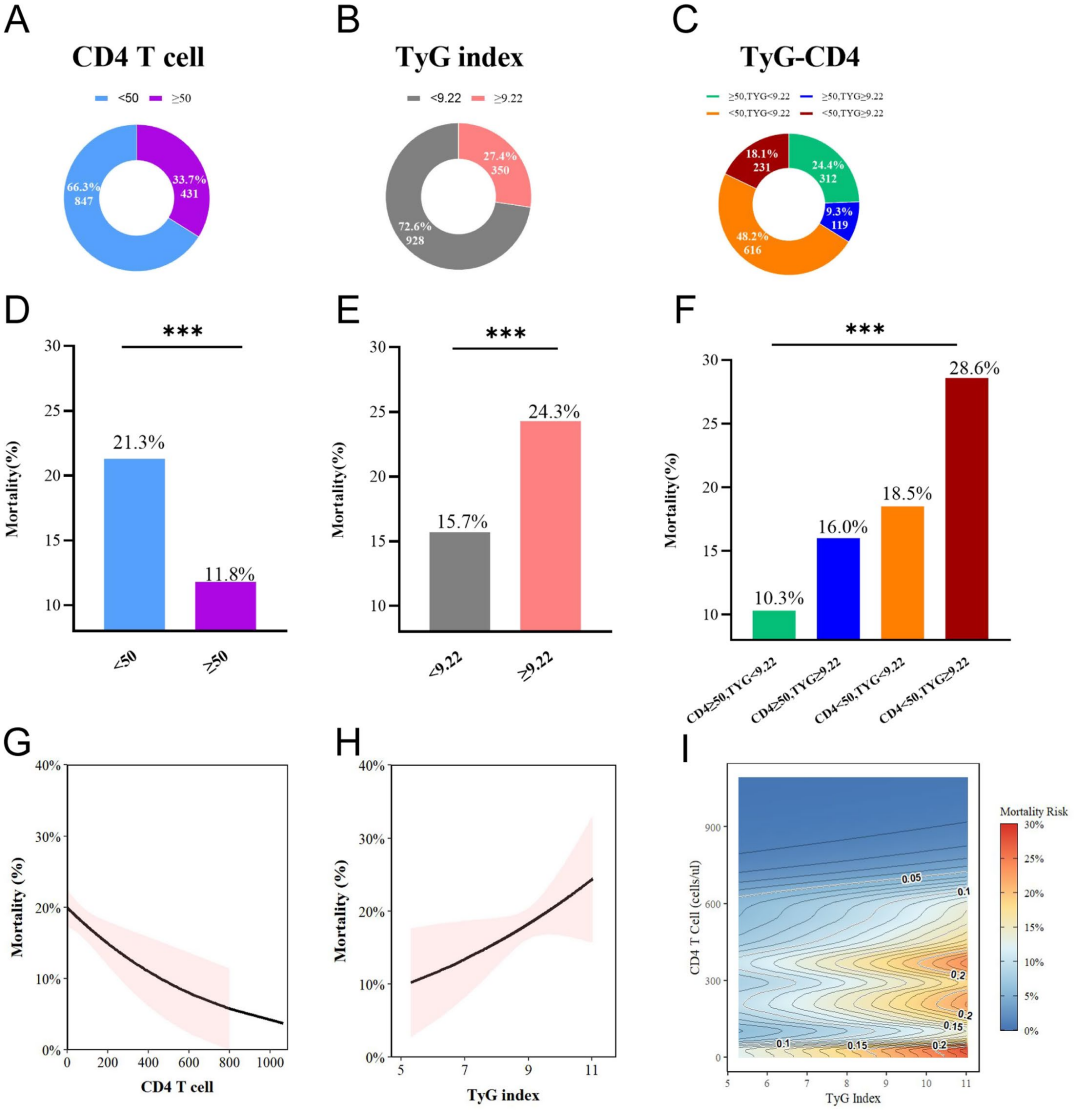
Association of CD4^+^ T cell count, TyG index, and TyG-CD4 with 28-day all-cause mortality in patients with HIV + sepsis. **(A-C)** Patient distribution: CD4 subgroups, TyG index subgroups, and combined TyG-CD4 subgroups. **(D-F)** Corresponding 28-day mortality rates: CD4 subgroups, TyG index subgroups, and combined TyG-CD4 subgroups. **(G,H)** Linear associations between mortality and **(G)** CD4^+^ T-cell count and **(H)** TyG index; solid lines are least-squares fits, and shaded areas are 95% CIs. **(I)** Contour map of predicted mortality generated from a model including both variables simultaneously; CD4 subgroups: (<50 vs. ≥ 50 cells/μL). TyG index subgroups: (<9.22 vs. ≥ 9.22). TyG-CD4 subgroups (CD4 ≥ 50&TyG < 9.22, CD4 ≥ 50&TyG ≥ 9.22, CD4 < 50&TyG < 9.22, and CD4 < 50&TyG ≥ 9.22).

**Figure 3:**
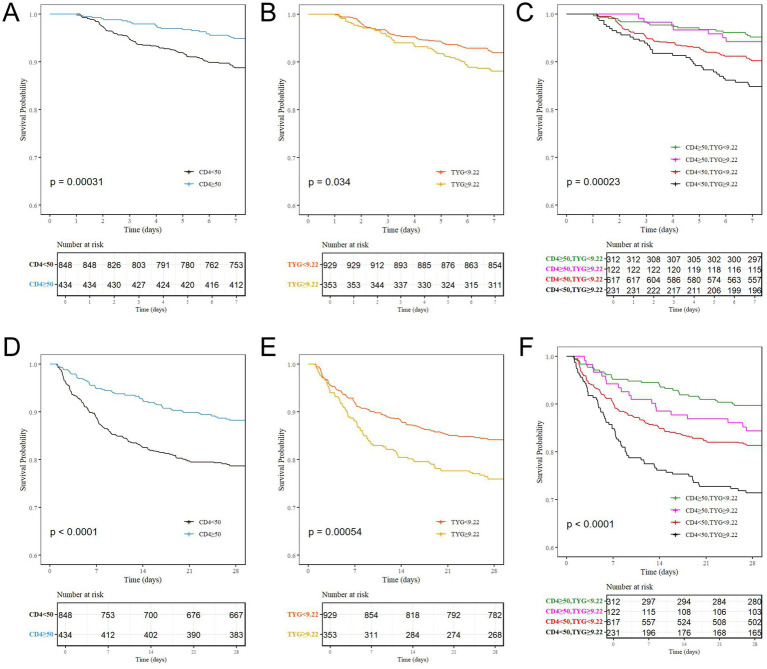
The Kaplan–Meier survival analysis stratified by CD4^+^ T cell count, TyG index, and TyG-CD4 with 28-day all-cause mortality in patients with HIV + sepsis. **(A-C)** Survival curves at 7 days stratified by CD4 subgroups, TyG index subgroups, and combined TyG-CD4 subgroups. **(D-F)** Survival curves at 28 days stratified by CD4 subgroups, TyG index subgroups, and combined TyG-CD4 subgroups. CD4 subgroups: (<50 vs. ≥ 50 cells/μL). TyG index subgroups (<9.22 vs. ≥ 9.22). TyG-CD4 subgroups (CD4 ≥ 50&TyG < 9.22, CD4 ≥ 50&TyG ≥ 9.22, CD4 < 50&TyG < 9.22, and CD4 < 50&TyG ≥ 9.22). TyG, triglyceride-glucose.

Overall, 28-day all-cause mortality differed according to TyG status (24.3% vs. 15.7%; *p* < 0.001) and CD4 status (21.3% vs. 11.8%; *p* < 0.001). Patients in the non-survivor group exhibited greater disease severity and multi-organ dysfunction, as evidenced by significantly higher SOFA scores, a markedly higher incidence of septic shock, and a substantially shorter hospital length of stay. Laboratory findings revealed lower CD4 counts (19 vs. 26 cells/μL), elevated triglyceride levels and TyG index, increased total bilirubin and creatinine concentrations, and reduced platelet counts (all *p* < 0.05), indicating a high-risk phenotype characterized by immunosuppression and metabolic dysregulation (Table 2). Mortality increased across the four combined strata, from 10.3% in the CD4 ≥ 50 &TyG < 9.22 group to 28.6% in the CD4 < 50 &TyG ≥ 9.22 group (*p* < 0.001). The same gradient was observed for 7-day and in-hospital deaths (Table 1, Figure 2). In Cox models (Table 3), relative to the reference group (CD4 ≥ 50 &TyG < 9.22), the adjusted hazard ratio (Model 3) for 28-day mortality in the CD4 < 50 &TyG ≥ 9.22 group was 2.82 (95% CI, 1.77–4.50; *p* < 0.001), with a significant trend across strata (*p* for trend <0.001). The Kaplan–Meier curves corroborated these findings: 7-day and 28-day cumulative mortality rates were highest in the combined high-risk group (14.2 and 29.1%, respectively; both log-rank *p* < 0.001) (Figure 3).

**Table 2:** Baseline characteristics of the survivor and non-survivor groups.

Variable	Total (*n* = 1,278)	Survivor (*n* = 1,047)	Non-survivor (*n* = 231)	*p*-value
Age, mean ± SD, years	43.8 ± 13.9	43.5 ± 13.8	45.5 ± 14.4	0.048
Male, *n* (%)	1,074 (84.0)	881 (84.1)	193 (83.5)	0.823
SOFA score, mean ± SD	5.3 ± 3.0	4.7 ± 2.7	7.7 ± 3.5	<0.001
Hospital length of stay, mean ± SD, days	26.5 ± 21.6	30.6 ± 21.7	8.1 ± 6.3	<0.001
ICU admission, *n* (%)	198 (15.5)	100 (9.6)	98 (42.4)	<0.001
Septic shock, *n* (%)	461 (36.1)	262 (25.0)	199 (86.1)	<0.001
Comorbidities				
Chronic kidney disease, *n* (%)	54 (4.2)	45 (4.3)	9 (3.9)	0.783
Liver cirrhosis, *n* (%)	68 (5.3)	50 (4.8)	18 (7.8)	0.064
COPD, *n* (%)	79 (6.2)	71 (6.8)	8 (3.5)	0.058
Diabetes, *n* (%)	82 (6.4)	64 (6.1)	18 (7.8)	0.346
Hypertension, *n* (%)	87 (6.8)	70 (6.7)	17 (7.4)	0.713
Coronary heart disease, *n* (%)	49 (3.8)	38 (3.6)	11 (4.8)	0.417
Opportunistic infections				
Talaromycosis, *n* (%)	468 (36.6)	380 (36.3)	88 (38.1)	0.607
*Pneumocystis pneumonia*, *n* (%)	219 (17.1)	174 (16.6)	45 (19.5)	0.296
Cryptococcosis, *n* (%)	92 (7.2)	83 (7.9)	9 (3.9)	0.032
Candidiasis, *n* (%)	410 (32.1)	337 (32.2)	73 (31.6)	0.863
Treatments				
Antiretroviral therapy, *n* (%)	433 (33.9)	368 (35.1)	65 (28.1)	0.042
Heparin use, *n* (%)	91 (7.1)	60 (5.7)	31 (13.4)	<0.001
Vasopressor use, *n* (%)	445 (34.8)	228 (21.8)	217 (93.9)	<0.001
Glucocorticoid use, *n* (%)	206 (16.1)	133 (12.7)	73 (31.6)	<0.001
Invasive mechanical ventilation, *n* (%)	227 (17.8)	108 (10.3)	119 (51.5)	<0.001
CRRT use, *n* (%)	92 (7.2)	34 (3.2)	58 (25.1)	<0.001
Laboratory values				
CD4 count, median (IQR), cells/μL	24.0 (8.0–82.8)	26.0 (8.0–92.0)	19.0 (7.0–45.0)	0.008
Glucose, median (IQR), mg/dL	102.6 (86.6–126.0)	102.8 (88.0–124.7)	102.4 (77.0–133.3)	0.278
Triglycerides, median (IQR), mg/dL	136.4 (99.2–191.3)	133.7 (99.2–182.9)	152.3 (97.9–236.5)	0.009
TyG index, median (IQR)	8.9 (8.5–9.3)	8.9 (8.5–9.2)	9.0 (8.5–9.4)	0.049
Total bilirubin, median (IQR), μmol/L	10.5 (6.9–19.4)	9.8 (6.7–16.1)	17.4 (8.9–40.1)	<0.001
Creatinine, median (IQR), μmol/L	73.0 (58.5–99.4)	72.0 (58.0–92.3)	83.0 (60.1–155.8)	<0.001
Platelets, median (IQR), ×10^3^/μL	136.5 (63.0–225.0)	150.0 (74.0–236.5)	80.0 (34.5–160.5)	<0.001
Lymphocytes, median (IQR), ×10^9^/L	0.5 (0.3–1.0)	0.5 (0.3–1.0)	0.5 (0.2–1.0)	0.203
White blood cells, median (IQR), ×10^9^/L	5.1 (3.2–8.1)	4.9 (3.1–7.7)	6.5 (3.6–10.2)	<0.001
Urea, median (IQR), mmol/L	5.1 (3.6–8.6)	4.8 (3.4–7.3)	8.4 (5.1–15.6)	<0.001
Monocytes, median (IQR), ×10^9^/L	0.2 (0.1–0.4)	0.2 (0.1–0.4)	0.2 (0.1–0.4)	0.049
Lactate dehydrogenase, median (IQR), U/L	369.5 (255.0–651.2)	334.0 (243.0–522.0)	715.0 (416.5–1713.5)	<0.001
Albumin, median (IQR), g/L	27.0 (22.2–32.2)	28.0 (23.0–33.0)	23.0 (19.0–28.0)	<0.001

TyG index, triglyceride-glucose index, SOFA, Sequential Organ Failure Assessment SCORE; ICU, intensive care unit; COPD, chronic obstructive pulmonary disease; CRRT, continuous renal replacement therapy.

**Table 3:** Hazard ratios and 95% confidence intervals for 7-day mortality, 28-day mortality, and hospital mortality based on TYG-CD4.

Outcome	Categories		Model 1			Model 2			Model 3		
	TYG-CD4	Events (%)	HR (95% CI)	*p*-value	*p* for trend	HR (95% CI)	*p*-value	*p* for trend	HR (95% CI)	*p*-value	*p* for trend
7 days mortality					<0.001			<0.001			<0.001
	CD4 ≥ 50, TYG < 9.22	15/312	refer			refer			refer		
	CD4 ≥ 50, TYG ≥ 9.22	7/119	1.22 (0.50–3.00)	0.66		1.25 (0.51–3.06)	0.632		1.00 (0.40–2.51)	0.993	
	CD4 < 50, TYG < 9.22	59/616	2.05 (1.16–3.61)	0.013		2.20 (1.24–3.92)	0.007		2.35 (1.25–4.42)	0.008	
	CD4 < 50, TYG ≥ 9.22	35/231	3.34 (1.82–6.12)	<0.001		3.57 (1.93–6.59)	<0.001		2.69 (1.37–5.30)	0.004	
28 days mortality					<0.001			<0.001			<0.001
	CD4 ≥ 50, TYG < 9.22	32/312	refer			refer			refer		
	CD4 ≥ 50, TYG ≥ 9.22	19/119	1.591 (0.902–2.807)	0.109		1.619 (0.918–2.858)	0.096		1.341 (0.750–2.395)	0.322	
	CD4 < 50, TYG < 9.22	114/616	1.912 (1.292–2.831)	0.001		2.117 (1.421–3.154)	<0.001		2.287 (1.485–3.521)	<0.001	
	CD4 < 50, TYG ≥ 9.22	66/231	3.153 (2.067–4.810)	<0.001		3.481 (2.268–5.342)	<0.001		2.820 (1.766–4.504)	<0.001	
Hospital mortality					<0.001			<0.001			<0.001
	CD4 ≥ 50, TYG < 9.22	58/312	refer			refer			refer		
	CD4 ≥ 50, TYG ≥ 9.22	25/119	1.387 (0.864–2.226)	0.17552		1.408 (0.877–2.260)	0.157045		1.043 (0.637–1.706)	0.868	
	CD4 < 50, TYG < 9.22	168/616	1.576 (1.163–2.137)	0.00338		1.817 (1.332–2.479)	<0.001		2.042 (1.453–2.868)	<0.001	
	CD4 < 50, TYG ≥ 9.22	84/231	2.328 (1.659–3.269)	<0.001		2.705 (1.915–3.821)	<0.001		2.398 (1.640–3.508)	<0.001	

Model 1: Unadjusted model.

Model 2: Adjusted for age and sex.

Model 3: Model 2 with additional adjustments for the following covariates: diabetes, hypertension, chronic kidney disease, liver cirrhosis, coronary heart disease, chronic obstructive pulmonary disease, Sequential Organ Failure Assessment score, antiretroviral therapy, talaromycosis, *Pneumocystis pneumonia*, cryptococcosis, and candidiasis.

HR, hazard ratio; CI, confidence interval.

### Feature selection and model discrimination

**Figure 4:**
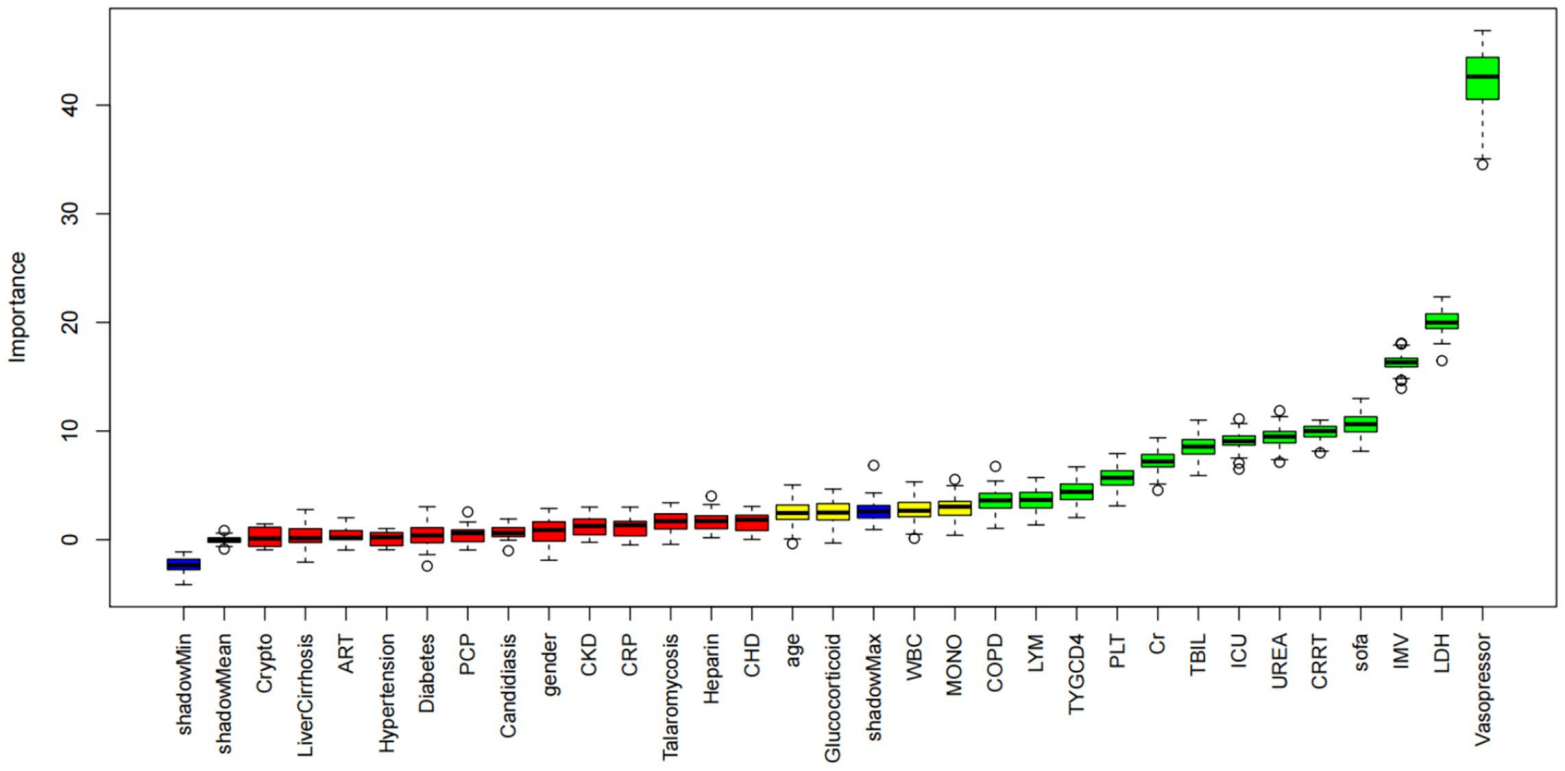
Feature selection based on the Boruta algorithm. The horizontal axis is the name of each variable, and the vertical axis is the *Z* value of each variable. The box plot shows the *Z* value of each variable during model calculation. The green boxes represent important variables, and the red boxes represent unimportant variables. ART, antiretroviral therapy; CKD, chronic kidney disease; CHD, coronary heart disease; COPD, chronic obstructive pulmonary disease; ICU, intensive care unit; IMV, invasive mechanical ventilation; Crypto, cryptococcosis; PCP, *Pneumocystis pneumonia*; TBIL, total bilirubin; Cr, creatinine; LDH, lactate dehydrogenase; LYM, lymphocytes; MONO, monocytes; PLT, platelet count; UREA, blood urea nitrogen; WBC, white blood cell count; ALB, albumin; SOFA, Sequential Organ Failure Assessment score; CRRT, continuous renal replacement therapy; Heparin, heparin anticoagulation therapy; Glucocorticoid, glucocorticoid therapy; Vasopressor, vasopressor therapy.

Boruta analysis identified the combined TyG-CD4 category as a key predictor of 28-day mortality (Figure 4), consistent with the results of Cox analysis. The AUC for the combined model was 0.605 (95% CI, 0.568–0.642), significantly exceeding those of TyG alone (0.541; *p* = 0.004) or CD4 alone (0.556; *p* = 0.002) (Table 4).

**Table 4:** Area under the receiver operating characteristic curve for 28-day all-cause mortality according to TyG index, CD4^+^ T-cell count, and TyG-CD4 in patients with HIV + sepsis.

Variable	AUC	Down (95% CI)	Up (95% CI)	AUC comparison	*p*
TYG	0.541	0.497	0.586	vs. TYG-CD4	0.004
CD4	0.556	0.516	0.595	vs. TYG-CD4	0.002
TYG-CD4	0.605	0.568	0.642		

HR, hazard ratio; CI, confidence interval.

### Subgroup analysis

**Figure 5:**
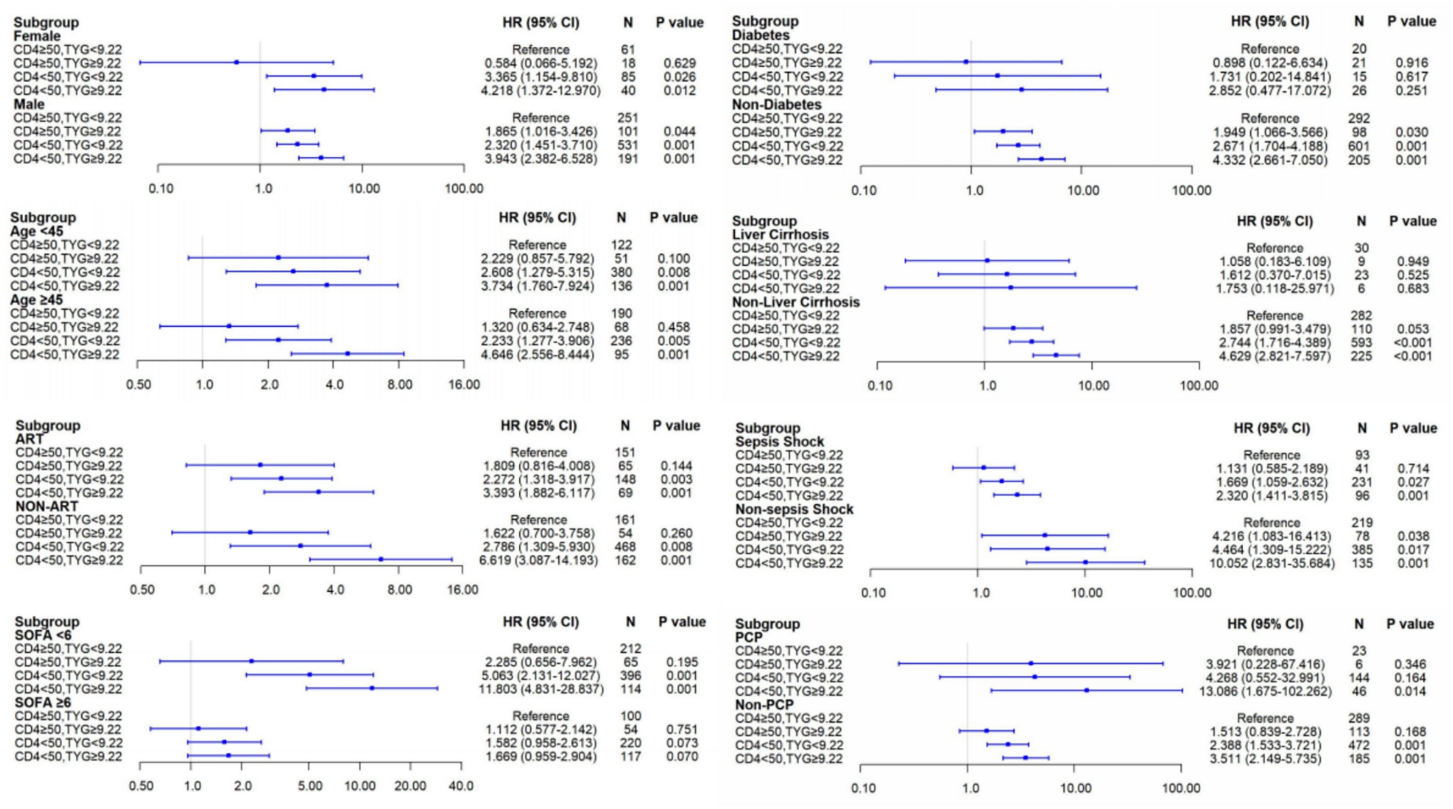
Forest plots of 28-day mortality risk stratified by TyG index and CD4^+^ T-cell count in HIV patients with sepsis. Subgroup analyses show adjusted hazard ratios (HRs) and 95% confidence intervals (CIs) for associations between TyG-CD4 joint stratification and 28-day mortality risk in HIV patients with sepsis. Analyses were stratified by: sex, diabetes status, age, liver cirrhosis,septic shock, SOFA score, and *Pneumocystis pneumonia* status. All models were adjusted for age, gender, hypertension, chronic kidney disease, liver cirrhosis, diabetes, coronary heart disease, chronic obstructive pulmonary disease, talaromycosis, *Pneumocystis pneumonia*, cryptococcosis, and candidiasis. The reference group (HR = 1.00) corresponds to the low-risk TyG-CD4 stratum (CD4 ≥ 50 cells/μL & TyG < 9.22). Square markers represent point estimates of HRs; horizontal lines indicate 95% CIs. Sample size (*N*) and *p*-values are shown for each subgroup. Dashed vertical line denotes no effect (HR = 1.0). CI, confidence interval; HR, hazard ratio; SOFA, Sequential Organ Failure Assessment; ART, antiretroviral therapy; PCP, *Pneumocystis pneumonia*.

After adjusting for age, sex, and comorbidities, a graded increase in mortality risk persisted across most prespecified subgroups (including sex, age, diabetes, antiretroviral therapy (ART) treatment, SOFA score, cirrhosis, septic shock, and PCP), with the high-CD4/low-TYG group as the reference (Figure 5). This elevated risk was most pronounced in the two low-CD4 subgroups. Overall, the combination of low CD4^+^ and high TYG levels consistently demonstrated an increased mortality risk. Notable exceptions were observed in women and patients with diabetes, where the association was not significant, likely because of the small subgroup sizes (*n* = 18 and *n* = 21, respectively).

### Cytokine profiling

**Figure 6:**
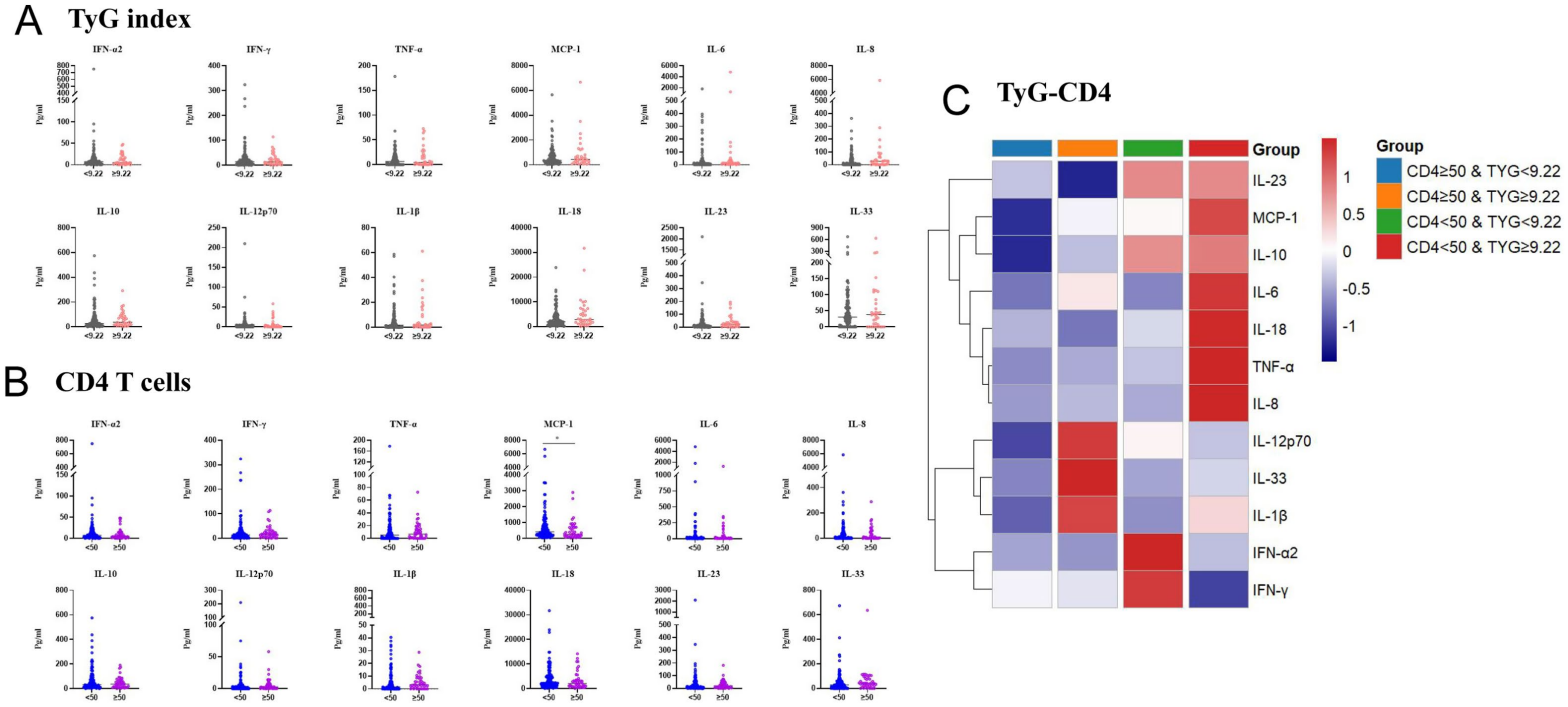
Cytokine profiles stratified by TyG index and CD4 T cell counts. Panels **(A)** and **(B)** display violin plots of 13 cytokines stratified by **(A)** TyG index and **(B)** CD4 T cell count, respectively. **(C)** Heatmap of cytokine expression across four TyG-CD4 strata. IFN, interferon; IL, interleukin; MCP-1, monocyte chemoattractant protein-1; TNF-α, tumor necrosis factor-α; TyG, triglyceride-glucose index.

Among the 155 participants, the clinical characteristics were comparable across the TyG-CD4 strata (Supplementary Table 6). After excluding IL-17A, because its levels were below the lower limit of quantification, the levels of 12 plasma cytokines were evaluated. Participants with advanced immunosuppression (CD4 < 50 cells/μL; *n* = 117) had significantly higher MCP-1 levels than those with CD4 ≥50 cells/μL [median (IQR), 448.5 (206.8–864.3) vs. 254.5 (121.4–681.2) pg/mL; *p* = 0.02] (Figure 6A). Cytokine levels did not differ significantly according to the TyG index alone; however, the TyG-high group (*n* = 34) demonstrated numerically higher IL-8, IL-10, and IL-18 levels, suggesting low-grade inflammation associated with insulin resistance (Figure 6B). In subgroup analyses stratified by CD4 count and TyG index, the group with low CD4 count and high TyG exhibited the broadest and highest cytokine upregulation, respectively. Despite an overall distinct cytokine profile in the high-risk group (CD4 < 50 & high-TyG) visualized by the *Z*-score heatmap (Figure 6C), further analysis revealed that, within the CD4 < 50 subgroup, a high TyG index was not associated with a global cytokine elevation. Instead, it was specifically linked to a pronounced upregulation of IL-18 (*p* = 0.009) and IL-23 (*p* = 0.025) compared to the low-TyG group (Supplementary Tables 7, 8). This finding indicates that, under profound immunosuppression, metabolic dysregulation may preferentially activate specific inflammatory pathways.

## Discussion

This is the first study to propose and validate a dual-biomarker stratification model that combines a surrogate for insulin resistance (the TyG index) with an immunodeficiency marker (CD4 T-cell count) to assess the mortality risk in HIV-associated sepsis. In a retrospective analysis of 1,278 patients, the composite model identified an extreme-risk subgroup (CD4 < 50 cells/μL and TyG ≥ 9.22) with a 28-day mortality of 28.6%. Although it demonstrates only a modest overall discriminatory ability, the immunometabolic stratification framework proposed in this study provides an important complementary perspective to traditional sepsis scoring systems such as the SOFA score and lays the conceptual groundwork for future exploration of more targeted risk stratification and management strategies.

In our cohort, the finding that 88.1% of patients had CD4 cell counts below 200 cells/μL, coupled with a high prevalence of talaromycosis (36.6%), indicates widespread and profound immunosuppression. Within this population, the “floor effect” of CD4 counts limits its ability to further discriminate differential prognostic outcomes, as its discriminatory power becomes saturated. Substantial evidence confirms that HIV-positive patients with sepsis face elevated mortality rates and a significant disease burden (14, 15). In current clinical practice, the CD4^+^ T lymphocyte count remains a cornerstone metric for assessing the degree of immunodeficiency, guiding disease staging, and informing management decisions in people living with HIV, with its prognostic value being well-established (16–18). Sepsis often induces metabolic dysregulation and IR, which engage in a bidirectional pathogenic cycle: systemic inflammation impairs insulin signaling, leading to IR, while IR-associated hyperinsulinemia promotes immunometabolic reprogramming and exacerbates inflammation (19–22). This disruption of immunometabolic homeostasis can compromise host defenses and worsen clinical outcomes (23, 24). The TyG index, a practical surrogate marker of IR, has been consistently validated for its prognostic value in sepsis (25–27). Furthermore, the combined use of the TyG index and CD4 count demonstrated statistically superior predictive value compared to either marker alone, enabling a more comprehensive assessment of patient vulnerability. Although the model’s discriminatory capacity limits its precision for individual-level prediction, it provides a valuable immunometabolic risk stratification perspective. Crucially, after adjusting for multiple covariates, including SOFA, the high-risk group (low CD4^+^ high TyG) maintained a 2.82-fold higher mortality risk (95% CI: 1.77–4.50) compared to the low-risk group (high CD4^+^ low TyG), indicating that this model provides prognostic information independent of acute organ dysfunction.

Sepsis is a highly heterogeneous syndrome, as evidenced by distinct clinical phenotypes and outcomes across patient subgroups (28–30). The TyG-CD4 stratification model proposed in this study is designed to address this heterogeneity by integrating two key dimensions of host vulnerability: immune dysfunction (CD4 count) and metabolic dysregulation (TyG index). This approach aims to identify a high-risk subpopulation characterized by a shared pathophysiology of profound immunosuppression coupled with metabolic disturbance. In our cohort, which had an overall 28-day mortality rate of 18.1%, the model effectively stratified patients into distinct risk groups: a high-risk group with a mortality rate of 28.6% and a low-risk group with a mortality rate of 10.3%. Our findings are consistent with the study of Cheng et al. (31), who reported broad defects in energy metabolism—evidenced by reduced ATP and NAD^+^ levels in leukocytes of patients with sepsis—a state termed “immunometabolic paralysis”. This aligns closely with our hypothesis that disruption of the immune–metabolic axis constitutes a core mechanism underlying high-risk phenotypes. Furthermore, the study by Hotchkiss et al. (32) highlighted that CD4^+^ T cell exhaustion and apoptosis are hallmark features of immunosuppression in lethal sepsis, further supporting the rationale and potential clinical value of integrating immune and metabolic dimensions for risk stratification.

Based on our previous research, HIV infection and associated metabolic disturbances may activate signaling pathways such as NLRP3 inflammasome and JNK, thereby interfering with insulin signaling (e.g., the PI3K/AKT pathway) and establishing a vicious cycle of chronic inflammation and insulin resistance (33–35). While our data do not directly test these specific pathways, the distinct inflammatory profile observed in our cohort—specifically, the significant association between a high TyG index and elevated IL-18 (a key cytokine downstream of NLRP3 inflammasome activation) in severely immunosuppressed patients—is consistent with this broader concept of immunometabolic crosstalk. This alignment between our associative findings and established pathophysiological frameworks generates the hypothesis that such mechanisms may be operative in this patient population, warranting direct investigation in future mechanistic studies. Notably, IR is commonly observed in patients with HIV, irrespective of combination antiretroviral therapy (cART) exposure (36), and profound immunosuppression may exacerbate IR by disrupting adipose tissue function, perturbing cytokine networks, and impairing hepatic metabolic homeostasis (37, 38). The tight coupling between immunosenescence and metabolic abnormalities in HIV suggests that metabolic interventions may be particularly effective in hosts with severe immune deficits (39). Our findings suggest that, in HIV-infected patients with sepsis, immune dysfunction and metabolic derangement act synergistically via defined inflammatory signaling pathways to shape a distinctive cytokine signature. Plasma MCP-1 and TNF-α are elevated in individuals with HIV; these are markers linked to monocyte activation and IR (40). Metabolic abnormalities, particularly in IR, may amplify these inflammatory responses and reinforce chronic low-grade immune activation. IL-6 and TNF-α can induce aberrant serine phosphorylation of IRS-1 through the JNK and NF-κB pathways, disrupting insulin signaling homeostasis (41, 42).

These mechanisms parallel “metaflammation” described in non-HIV populations but manifest in immunocompromised hosts as a more complex, biphasic state in which immunosuppression coexists with hyperinflammation. Following immune suppression, proinflammatory mediators such as TNF-α and IL-6 remain persistently elevated, while anti-inflammatory feedback (e.g., IL-10 and TGF-β) may be concurrently upregulated, an apparent “paradoxical immune activation” that has been discussed in HIV-related inflammation (43). In contrast, several mucosal/antiviral cytokines (e.g., IL-17 and IL-22) depend more directly on CD4^+^ T cell abundance and are relatively uncoupled from the metabolic status, indicating that dysglycemia and dyslipidemia do not uniformly affect all immune pathways (33, 44). Consistent with this biology, chronic low-grade inflammation in HIV—characterized by sustained elevations in IL-6 and TNF-α—perturbs insulin signaling and promotes IR via JNK and NF-κB activation (34). Sepsis-induced IR and secondary glucolipotoxicity can aggravate T-cell exhaustion, perpetuating a vicious cycle of immunologic and metabolic dysfunction; although described in non-HIV sepsis, these processes are plausibly more deleterious in HIV-associated sepsis (35). Moreover, in HIV cohorts, the TyG index correlates with CD8^+^ T-cell expansion, a lower CD4/CD8 ratio, and immunosenescent phenotypes, providing a mechanistic rationale for the uncontrolled inflammation observed in patients with profound CD4 depletion and a high metabolic load (39). Furthermore, while this observational study robustly demonstrates a significant association between TyG-CD4 stratification and sepsis outcomes, its design inherently precludes the definitive establishment of causality. The target trial emulation framework has emerged as a rigorous methodology widely adopted in observational research to address this limitation and evaluate potential causal effects (45, 46). Our study provides an ideal platform to apply this framework, utilizing advanced causal inference methods such as inverse probability of treatment weighting and g-computation to emulate a hypothetical randomized trial comparing outcomes between a management strategy guided by TyG-CD4 risk stratification and standard care.

### Clinical implications

Our findings support the incorporation of metabolic profiling into the routine assessment of critically ill patients with HIV, especially in resource-limited settings. Since the TyG-CD4 schema relies on glucose and triglyceride values that are already obtained in standard care, it offers a low-cost adjunct to CD4-based evaluation and enables rapid recognition of the highest-risk patients—particularly those with concurrent immunodeficiency and metabolic dysregulation—who may not be identified by CD4 alone. Although the model demonstrates moderate discriminative performance, its potential value lies in enabling early risk stratification using readily obtainable clinical parameters. In practice, it should be regarded as a useful complement to—rather than a replacement for—established severity scores such as the SOFA score. The composite stratification model developed in this study serves primarily as a hypothesis-generating framework for future investigation. By identifying distinct risk phenotypes, this study suggests the presence of divergent pathophysiological mechanisms across patient subgroups, thereby providing a biological rationale for exploring individualized management strategies. Future prospective implementation studies are warranted to assess their real-world clinical utility and impact on patient outcomes.

### Limitations

This study has several important limitations. First, its retrospective, single-center design and the unique cohort characteristics may limit the generalizability of the findings. Second, the lack of data on key covariates such as adherence to antiretroviral therapy, baseline disease load information, and the use of glucose- or lipid-lowering agents raises the possibility of residual confounding. The TyG index serves as a surrogate marker of insulin resistance and cannot directly capture detailed insulin dynamics; furthermore, stratification based on the Youden index carries a potential risk of overfitting. In addition, the discriminative ability of the combined TyG-CD4 model was modest, indicating that it is currently more suitable for risk stratification at the group level rather than for precise individual prognosis prediction. Therefore, the proposed TyG-CD4 stratification framework should be regarded as preliminary and hypothesis-generating. Its present value lies primarily in highlighting a distinct dimension of risk—“chronic immunometabolic vulnerability”—and in offering an early, readily accessible risk perspective for future research. The true clinical utility of this model will ultimately depend on rigorous validation in prospective studies that incorporate viral load and detailed treatment information.

### Future directions

It is necessary to conduct a multicenter prospective validation of the stratified model, incorporating additional immunometabolic markers, with viral load and detailed ART information included as covariates for further evaluation. The immediate priority is to conduct external validation of the stratification model in cohorts with differing epidemiological profiles to evaluate its generalizability and robustness. Subsequent efforts should focus on model refinement, for instance, by incorporating multidimensional immunometabolic biomarkers and tracking their dynamic changes to improve discriminatory performance. In terms of clinical utility, future studies must critically assess the incremental prognostic value and risk reclassification ability of the TyG-CD4 framework over established standards such as the SOFA score. Ultimately, these initiatives should culminate in translational research designed to evaluate a combined strategy of immune reconstitution and metabolic modulation for the “dual-high-risk” phenotype (low CD4/high TyG), systematically examining the feasibility, implementation fidelity, and cost-effectiveness of this precision approach in real-world settings.

## Conclusion

In patients with HIV-associated sepsis, the CD4 T-cell count and TyG index not only effectively distinguish clinical phenotypes with distinct prognostic outcomes, but their combination also serves as a valuable prognostic tool in this population. Furthermore, different risk strata exhibit unique inflammatory signatures, suggesting divergent underlying mechanisms of host immune regulation.

## Data Availability

The raw data supporting the conclusions of this article will be made available by the authors, without undue reservation.

## Footnotes

1
https://legendplex.qognit.com/

## Glossary

AUC: Area under the curve
ART: Antiretroviral therapy
CD4-TyG: Combined CD4 count and triglyceride-glucose index
Cox: Cox proportional hazards model
CRP: C-reactive protein
HIV: Human immunodeficiency virus
IFN-α2: Interferon alpha 2
IFN-γ: Interferon gamma
IL-1β: Interleukin 1 beta
IL-6: Interleukin 6
IL-8: Interleukin 8
IL-10: Interleukin 10
IL-12p70: Interleukin 12 p70
IL-17A: Interleukin 17A
IL-18: Interleukin 18
IL-23: Interleukin 23
IL-33: Interleukin 33
IR: Insulin resistance
LDH: Lactate dehydrogenase
MCP-1: Monocyte chemoattractant protein-1
PCP: *Pneumocystis pneumonia*
PLWH: People living with HIV
ROC: Receiver operating characteristic curve
SOFA: Sequential Organ Failure Assessment
TNF-α: Tumor necrosis factor alpha
TyG: Triglyceride-glucose index
