# Supplementary material for: Prognostic value of combined stratification using TyG index and CD4+ T cell count for 28-day all-cause mortality risk in patients with HIV infection and sepsis: a retrospective cohort study
Source: Front Med (Lausanne). 2026 Jan 12;12:1688334. doi: 10.3389/fmed.2025.1688334 (PMC12832338; doi:10.3389/fmed.2025.1688334)
Supplement: Supplementary file 1 [file Supplementary_file_1.docx]

**Supplementary Table1.Diagnostic Performance of the TyG Index for Identifying High 28‑Day Mortality Risk in patients With HIV+sepsis.**

| Variable | Cutoff | Sensitivity | Specificity | AUC | Youden_Index | Number_Events | Number_NonEvents |
| --- | --- | --- | --- | --- | --- | --- | --- |
| TyG index | 9.2244 | 0.3648 | 0.7474 | 0.5366 | 0.1122 | 233 | 1057 |

Cutoff selected at the maximal Youden index on the receiver operating characteristic curve generated from a multivariable logistic regression model.

**Supplementary Table 2. Assessment of Multicollinearity Among Candidate Covariates in the Mortality Model.**

| Variable | VIF |
| --- | --- |
| Age | 1.44 |
| Male | 1.04 |
| SOFA score | 2.19 |
| Hospital length of stay | 1.15 |
| ICU admission | 2.81 |
| Septic shock | 2.43 |
| Chronic kidney disease | 1.44 |
| Liver cirrhosis | 1.14 |
| COPD | 1.14 |
| Diabetes | 1.25 |
| Hypertension | 1.29 |
| Coronary heart disease | 1.11 |
| Talaromycosis | 1.37 |
| Pneumocystis pneumonia | 1.17 |
| Cryptococcosis | 1.08 |
| Candidiasis | 1.12 |
| Antiretroviral therapy | 1.13 |
| Heparin use | 1.30 |
| Vasopressor use | 2.84 |
| Glucocorticoid use | 1.48 |
| Invasive mechanical ventilation | 2.35 |
| CRRT use | 1.85 |
| CD4 count | 1.53 |
| Glucose | 1.18 |
| Triglycerides | 1.14 |
| Total bilirubin | 1.30 |
| Creatinine | 3.25 |
| Platelets | 1.51 |
| Lymphocytes | 1.52 |
| White blood cells | 1.85 |
| Urea | 3.18 |
| Monocytes | 1.91 |
| Lactate dehydrogenase | 1.63 |
| Albumin | 1.44 |

VIF values > 5 are generally considered to indicate moderate multicollinearity, and values > 10 indicate severe multicollinearity. None of the covariates exceeded these thresholds in the current model.

**Abbreviations:** COPD, chronic obstructive pulmonary disease; ICU, intensive care unit; SOFA, Sequential Organ Failure Assessment; VIF, variance inflation factor.

**Supplementary Table 3. Frequency of Missing Baseline Laboratory Measurements.**

| Variable | Missing_Count | Missing_Percent（%） |
| --- | --- | --- |
| CD4 count | 52 | 4.07 |
| Glucose | 44 | 3.44 |
| Triglycerides | 70 | 5.48 |
| Total bilirubin | 35 | 2.74 |
| Creatinine | 22 | 1.72 |
| Platelets | 7 | 0.55 |
| Lymphocytes | 4 | 0.31 |
| White blood cells | 4 | 0.31 |
| Urea | 46 | 3.6 |
| Monocytes | 7 | 0.55 |
| Lactate dehydrogenase | 51 | 3.99 |
| Albumin | 78 | 6.1 |

Percentages are calculated as (number of missing observations ÷ total analytic cohort) × 100. The total cohort size was N = 1 278.

**Supplementary Table4 Baseline characteristics of the study population according to the TyG index**

| ​​Variable​​ | ​​Total (n=1278)​​ | ​​TYG<9.22 (n=928)​​ | ​​TYG≥9.22 (n=350)​​ | ​​p value​​ |
| --- | --- | --- | --- | --- |
| Age, mean ± SD, y | 43.8 ± 13.9 | 43.9 ± 14.3 | 43.8 ± 12.7 | 0.975 |
| Male sex, n (%) | 1074 (84.0) | 782 (84.3) | 292 (83.4) | 0.715 |
| SOFA score, mean ± SD | 5.8 ± 3.5 | 5.5 ± 3.3 | 6.7 ± 3.7 | <0.001 |
| Hospital length of stay, mean ± SD, d | 26.5 ± 21.6 | 27.6 ± 22.2 | 23.8 ± 19.7 | 0.005 |
| 7-day mortality, n (%) | 116 (9.1) | 74 (8.0) | 42 (12.0) | 0.025 |
| 28-day mortality, n (%) | 231 (18.1) | 146 (15.7) | 85 (24.3) | <0.001 |
| In-hospital mortality, n (%) | 335 (26.2) | 226 (24.4) | 109 (31.1) | 0.014 |
| ICU admission, n (%) | 198 (15.5) | 123 (13.3) | 75 (21.4) | <0.001 |
| Septic shock, n (%) | 461 (36.1) | 324 (34.9) | 137 (39.1) | 0.16 |
| ​​Comorbidities​​ |  |  |  |  |
| Chronic kidney disease, n (%) | 54 (4.2) | 35 (3.8) | 19 (5.4) | 0.189 |
| Liver cirrhosis, n (%) | 68 (5.3) | 53 (5.7) | 15 (4.3) | 0.311 |
| COPD, n (%) | 79 (6.2) | 59 (6.4) | 20 (5.7) | 0.67 |
| Diabetes mellitus, n (%) | 82 (6.4) | 35 (3.8) | 47 (13.4) | <0.001 |
| Hypertension, n (%) | 87 (6.8) | 55 (5.9) | 32 (9.1) | 0.042 |
| Coronary heart disease, n (%) | 49 (3.8) | 35 (3.8) | 14 (4.0) | 0.85 |
| ​​Opportunistic Infections​​ |  |  |  |  |
| Talaromycosis, n (%) | 468 (36.6) | 334 (36.0) | 134 (38.3) | 0.448 |
| Pneumocystis pneumonia, n (%) | 219 (17.1) | 167 (18.0) | 52 (14.9) | 0.184 |
| Cryptococcosis, n (%) | 92 (7.2) | 68 (7.3) | 24 (6.9) | 0.772 |
| Candidiasis, n (%) | 410 (32.1) | 295 (31.8) | 115 (32.9) | 0.715 |
| ​​Treatments​​ |  |  |  |  |
| Antiretroviral therapy, n (%) | 433 (33.9) | 299 (32.2) | 134 (38.3) | 0.041 |
| Heparin use, n (%) | 91 (7.1) | 56 (6.0) | 35 (10.0) | 0.014 |
| Vasopressor use, n (%) | 445 (34.8) | 310 (33.4) | 135 (38.6) | 0.084 |
| Glucocorticoid use, n (%) | 206 (16.1) | 136 (14.7) | 70 (20.0) | 0.02 |
| Invasive mechanical ventilation, n (%) | 227 (17.8) | 147 (15.8) | 80 (22.9) | 0.003 |
| CRRT use, n (%) | 92 (7.2) | 52 (5.6) | 40 (11.4) | <0.001 |
| ​​Laboratory Values​​ |  |  |  |  |
| CD4 count, median (IQR), cells/μL | 24.0 (8.0-82.8) | 24.0 (7.0-84.2) | 26.5 (9.0-81.2) | 0.313 |
| Glucose, median (IQR), mg/dL | 102.6 (86.6-126.0) | 97.2 (82.8-115.0) | 128.7 (102.8-170.6) | <0.001 |
| Triglycerides, median (IQR), mg/dL | 136.4 (99.2-191.3) | 117.8 (90.3-150.6) | 231.2 (177.1-306.5) | <0.001 |
| TyG index, median (IQR) | 8.9 (8.5-9.3) | 8.7 (8.4-8.9) | 9.6 (9.4-9.9) | <0.001 |
| Total bilirubin, median (IQR), μmol/L | 10.5 (6.9-19.4) | 9.8 (6.7-16.8) | 13.0 (7.7-26.2) | <0.001 |
| Creatinine, median (IQR), μmol/L | 73.0 (58.5-99.4) | 71.0 (56.9-89.0) | 82.8 (63.7-138.9) | <0.001 |
| Platelets, median (IQR), ×10³/μL | 136.5 (63.0-225.0) | 148.5 (68.8-237.2) | 107.0 (52.0-183.5) | <0.001 |
| Lymphocytes, median (IQR), ×10⁹/L | 0.5 (0.3-1.0) | 0.5 (0.3-1.0) | 0.5 (0.3-1.0) | 0.36 |
| White blood cells, median (IQR), ×10⁹/L | 5.1 (3.2-8.1) | 5.0 (3.2-8.0) | 5.3 (3.2-8.5) | 0.508 |
| Urea, median (IQR), mmol/L | 5.1 (3.6-8.6) | 4.8 (3.5-7.3) | 6.9 (4.1-12.5) | <0.001 |
| Monocytes, median (IQR), ×10⁹/L | 0.2 (0.1-0.4) | 0.2 (0.1-0.4) | 0.2 (0.1-0.4) | 0.01 |
| Lactate dehydrogenase, median (IQR), U/L | 369.5 (255.0-651.2) | 343.0 (248.0-575.2) | 456.5 (289.0-913.0) | <0.001 |
| Albumin, median (IQR), g/L | 27.0 (22.2-32.2) | 27.0 (22.2-32.6) | 26.6 (22.1-31.1) | 0.196 |

**Abbreviations:** TyG:  index triglyceride-glucose index,SOFA: Sequential Organ Failure Assessment Score.ICU: Intensive Care Unit.COPD: Chronic Obstructive Pulmonary Disease.CRRT: Continuous Renal Replacement Therapy

**Supplementary Table5 Baseline characteristics of the study population according to the CD4+ T Cell count**

| ​​Variable​​ | ​​Total (n=1278)​​ | ​​CD4<50 (n=847)​​ | ​​CD4≥50 (n=431)​​ | ​​p value​​ |
| --- | --- | --- | --- | --- |
| Age, mean ± SD, y | 43.8 ± 13.9 | 41.6 ± 13.1 | 48.2 ± 14.4 | <0.001 |
| Male, n (%) | 1074 (84.0) | 722 (85.2) | 352 (81.7) | 0.099 |
| SOFA score, mean ± SD | 5.8 ± 3.5 | 6.0 ± 3.6 | 5.5 ± 3.2 | 0.012 |
| Hospital length of stay, mean ± SD, d | 26.5 ± 21.6 | 26.6 ± 20.8 | 26.5 ± 23.2 | 0.975 |
| 7-day mortality, n (%) | 116 (9.1) | 94 (11.1) | 22 (5.1) | <0.001 |
| 28-day mortality, n (%) | 231 (18.1) | 180 (21.3) | 51 (11.8) | <0.001 |
| In-hospital mortality, n (%) | 335 (26.2) | 252 (29.8) | 83 (19.3) | <0.001 |
| ICU admission, n (%) | 198 (15.5) | 145 (17.1) | 53 (12.3) | 0.024 |
| Septic shock, n (%) | 461 (36.1) | 327 (38.6) | 134 (31.1) | 0.008 |
| ​​Comorbidities​​ |  |  |  |  |
| Chronic kidney disease, n (%) | 54 (4.2) | 20 (2.4) | 34 (7.9) | <0.001 |
| Liver cirrhosis, n (%) | 68 (5.3) | 29 (3.4) | 39 (9.0) | <0.001 |
| COPD, n (%) | 79 (6.2) | 45 (5.3) | 34 (7.9) | 0.071 |
| Diabetes mellitus, n (%) | 82 (6.4) | 41 (4.8) | 41 (9.5) | 0.001 |
| Hypertension, n (%) | 87 (6.8) | 28 (3.3) | 59 (13.7) | <0.001 |
| Coronary heart disease, n (%) | 49 (3.8) | 22 (2.6) | 27 (6.3) | 0.001 |
| ​​Opportunistic Infections​​ |  |  |  |  |
| Talaromycosis, n (%) | 468 (36.6) | 418 (49.4) | 50 (11.6) | <0.001 |
| Pneumocystis pneumonia, n (%) | 219 (17.1) | 190 (22.4) | 29 (6.7) | <0.001 |
| Cryptococcosis, n (%) | 92 (7.2) | 69 (8.1) | 23 (5.3) | 0.066 |
| Candidiasis, n (%) | 410 (32.1) | 322 (38.0) | 88 (20.4) | <0.001 |
| ​​Treatments​​ |  |  |  |  |
| Antiretroviral therapy, n (%) | 433 (33.9) | 217 (25.6) | 216 (50.1) | <0.001 |
| Heparin use, n (%) | 91 (7.1) | 50 (5.9) | 41 (9.5) | 0.018 |
| Vasopressor use, n (%) | 445 (34.8) | 331 (39.1) | 114 (26.5) | <0.001 |
| Glucocorticoid use, n (%) | 206 (16.1) | 159 (18.8) | 47 (10.9) | <0.001 |
| Invasive mechanical ventilation, n (%) | 227 (17.8) | 174 (20.5) | 53 (12.3) | <0.001 |
| CRRT use, n (%) | 92 (7.2) | 65 (7.7) | 27 (6.3) | 0.357 |
| ​​Laboratory Values​​ |  |  |  |  |
| CD4 count, median (IQR), cells/μL | 24.0 (8.0-82.8) | 11.0 (5.0-24.0) | 140.0 (82.0-265.0) | <0.001 |
| Glucose, median (IQR), mg/dL | 102.6 (86.6-126.0) | 102.1 (85.7-125.5) | 104.9 (88.2-129.1) | 0.054 |
| Triglycerides, median (IQR), mg/dL | 136.4 (99.2-191.3) | 139.9 (102.7-191.3) | 125.8 (91.7-191.3) | 0.008 |
| TyG index, median (IQR) | 8.9 (8.5-9.3) | 8.9 (8.5-9.3) | 8.8 (8.4-9.3) | 0.237 |
| Total bilirubin, median (IQR), μmol/L | 10.5 (6.9-19.4) | 10.8 (7.2-20.5) | 9.6 (6.4-17.3) | 0.004 |
| Creatinine, median (IQR), μmol/L | 73.0 (58.5-99.4) | 70.0 (57.0-91.0) | 80.0 (62.9-112.0) | <0.001 |
| Platelets, median (IQR), ×10⁹/L | 136.5 (63.0-225.0) | 116.0 (52.0-204.5) | 173.0 (91.5-264.5) | <0.001 |
| Lymphocytes, median (IQR), ×10⁹/L | 0.5 (0.3-1.0) | 0.4 (0.2-0.7) | 1.0 (0.6-1.4) | <0.001 |
| White blood cells, median (IQR), ×10⁹/L | 5.1 (3.2-8.1) | 4.5 (2.9-7.2) | 6.5 (4.1-10.0) | <0.001 |
| Urea, median (IQR), mmol/L | 5.1 (3.6-8.6) | 5.0 (3.5-8.1) | 5.5 (3.9-9.6) | 0.013 |
| Monocytes, median (IQR), ×10⁹/L | 0.2 (0.1-0.4) | 0.2 (0.1-0.3) | 0.4 (0.2-0.6) | <0.001 |
| Lactate dehydrogenase, median (IQR), U/L | 369.5 (255.0-651.2) | 424.0 (279.0-745.0) | 292.0 (224.0-441.5) | <0.001 |
| Albumin, median (IQR), g/L | 27.0 (22.2-32.2) | 25.7 (21.0-30.0) | 30.2 (26.0-35.0) | <0.001 |

**Abbreviations:** TyG index :triglyceride-glucose index,SOFA: Sequential Organ Failure Assessment Score.ICU: Intensive Care Unit.

COPD: Chronic Obstructive Pulmonary Disease.CRRT: Continuous Renal Replacement Therapy

**Supplementary Table 6 Baseline characteristics of the Plasma Cytokine study population according to the TYG-CD4.**

| Variable | Total (n = 155) | ​​CD4≥50 & TYG<9.22 (n = 26) | CD4≥50 & TYG≥9.22 (n = 12) | ​​CD4<50 & TYG<9.22(n = 95) | ​​CD4<50 & TYG≥9.22(n = 22) | p |
| --- | --- | --- | --- | --- | --- | --- |
| Age, Mean ± SD | 37.8 ± 11.7 | 34.8 ± 9.9 | 41.6 ± 14.0 | 38.6 ± 12.3 | 35.5 ± 8.7 | 0.229 |
| Male, n (%) | 133 (85.8) | 21 (80.8) | 11 (91.7) | 85 (89.5) | 16 (72.7) | 0.168 |
| SOFA score, mean ± SD | 4.3 ± 2.3 | 4.7 ± 2.6 | 4.2 ± 2.5 | 4.0 ± 2.1 | 4.8 ± 2.8 | 0.377 |
| Hospital length of stay, mean ± SD, d | 27.9 (15.2, 39.5) | 27.3 (7.2, 40.4) | 29.3 (7.9, 33.8) | 28.8 (19.9, 40.9) | 24.8 (10.3, 33.0) | 0.386 |
| ICU admission, n (%) | 11 ( 7.1) | 2 (7.7) | 2 (16.7) | 5 (5.3) | 2 (9.1) | 0.35 |
| Septic shock, n (%) | 23 (14.8) | 3 (11.5) | 3 (25) | 12 (12.6) | 5 (22.7) | 0.39 |
| ​​Comorbidities​​ |  |  |  |  |  |  |
| Chronic kidney disease, n (%) | 1 ( 0.6) | 0 (0) | 0 (0) | 0 (0) | 1 (4.5) | 0.21 |
| Liver cirrhosis, n (%) | 4 ( 2.6) | 1 (3.8) | 0 (0) | 2 (2.1) | 1 (4.5) | 0.574 |
| COPD, n (%) | 10 ( 6.5) | 2 (7.7) | 1 (8.3) | 7 (7.4) | 0 (0) | 0.646 |
| Diabetes, n (%) | 6 ( 3.9) | 1 (3.8) | 0 (0) | 2 (2.1) | 3 (13.6) | 0.086 |
| Hypertension, n (%) | 5 ( 3.2) | 0 (0) | 1 (8.3) | 2 (2.1) | 2 (9.1) | 0.164 |
| Coronary heart disease, n (%) | 0 ( 0.0) | 0 (0) | 0 (0) | 0 (0) | 0 (0) | 1 |
| ​​Opportunistic Infections​​ |  |  |  |  |  |  |
| Talaromycosis, n (%) | 66 (42.6) | 6 (23.1) | 2 (16.7) | 46 (48.4) | 12 (54.5) | 0.019 |
| Pneumocystis pneumonia, n (%) | 39 (25.2) | 8 (30.8) | 2 (16.7) | 25 (26.3) | 4 (18.2) | 0.733 |
| Cryptococcosis, n (%) | 4 ( 2.6) | 0 (0) | 1 (8.3) | 2 (2.1) | 1 (4.5) | 0.281 |
| Candidiasis, n (%) | 70 (45.2) | 10 (38.5) | 3 (25) | 45 (47.4) | 12 (54.5) | 0.333 |
| ​​Treatments​​ |  |  |  |  |  |  |
| Antiretroviral therapy, n (%) | 29 (18.7) | 8 (30.8) | 2 (16.7) | 16 (16.8) | 3 (13.6) | 0.421 |
| Heparin, n (%) | 4 ( 2.6) | 2 (7.7) | 0 (0) | 2 (2.1) | 0 (0) | 0.405 |
| Vasopressor, n (%) | 20 (12.9) | 2 (7.7) | 4 (33.3) | 9 (9.5) | 5 (22.7) | 0.049 |
| Glucocorticoid use, n (%) | 17 (11.0) | 3 (11.5) | 1 (8.3) | 8 (8.4) | 5 (22.7) | 0.255 |
| Invasive mechanical ventilation, n (%) | 13 ( 8.4) | 2 (7.7) | 3 (25) | 5 (5.3) | 3 (13.6) | 0.069 |
| CRRT use, n (%) | 5 ( 3.2) | 1 (3.8) | 2 (16.7) | 1 (1.1) | 1 (4.5) | 0.023 |

**Abbreviations:** TYG-CD4:  index triglyceride-glucose Index and CD4+ T‑Cell Count,SOFA: Sequential Organ Failure Assessment Score.ICU: Intensive Care Unit.COPD: Chronic Obstructive Pulmonary Disease.CRRT: Continuous Renal Replacement Therapy

**Supplementary Table 7.Plasma Cytokine Levels in HIV+sepsis Patients Stratified by TYG-CD4**

| Variables | Total  (n = 155) | ​​CD4≥50 & TYG<9.22 (n = 26)​​ | ​​CD4≥50 & TYG≥9.22 (n = 12)​ | ​​CD4<50 & TYG<9.22 (n = 95) | ​​CD4<50 & TYG≥9.22 (n = 22)​ | p |
| --- | --- | --- | --- | --- | --- | --- |
| IL.1b, Median (IQR) | 1.7 (0.0, 5.7) | 3.3 (1.3, 8.4) | 2.3 (0.9, 6.2) | 1.3 (0.0, 4.8) | 1.4 (0.0, 9.8) | 0.269 |
| IFN.a2, Median (IQR) | 7.0 (2.9, 15.1) | 6.7 (1.8, 14.7) | 3.9 (2.2, 7.0) | 7.2 (3.2, 13.8) | 10.1 (2.9, 20.3) | 0.583 |
| IFN.y, Median (IQR) | 14.2 (6.5, 28.8) | 18.5 (9.8, 30.1) | 12.7 (5.9, 28.6) | 14.0 (6.1, 27.9) | 13.1 (7.2, 27.7) | 0.857 |
| TNF.a, Median (IQR) | 5.4 (0.0, 17.2) | 7.4 (1.7, 17.0) | 1.0 (0.0, 12.9) | 5.2 (0.0, 14.5) | 5.4 (1.3, 26.3) | 0.381 |
| MCP.1, Median (IQR) | 379.4 (189.6, 784.4) | 204.3 (119.3, 465.1) | 358.7 (165.3, 904.8) | 419.6 (211.6, 867.5) | 452.3 (196.8, 682.9) | 0.066 |
| IL.6, Median (IQR) | 9.2 (3.6, 26.9) | 8.2 (5.0, 24.6) | 15.9 (8.2, 64.4) | 7.7 (3.4, 26.9) | 11.7 (4.4, 19.5) | 0.509 |
| IL.8, Median (IQR) | 9.9 (0.3, 39.8) | 6.7 (0.0, 19.2) | 31.6 (4.6, 65.1) | 9.2 (0.5, 41.3) | 12.6 (3.1, 32.0) | 0.37 |
| IL.10, Median (IQR) | 27.9 (14.8, 71.4) | 28.4 (10.4, 61.0) | 26.2 (17.4, 71.9) | 27.3 (15.1, 69.4) | 45.0 (18.4, 91.8) | 0.511 |
| IL.12p70, Median (IQR) | 2.0 (0.0, 5.9) | 3.6 (0.3, 6.6) | 1.7 (0.0, 10.4) | 1.6 (0.0, 4.9) | 0.9 (0.0, 5.3) | 0.582 |
| IL.18, Median (IQR) | 2129.1 (1120.6, 4611.8) | 1747.2 (738.5, 3420.4) | 1898.2 (1184.0, 2735.6) | 1991.3 (1166.0, 4357.5) | 4811.6 (1938.0, 8332.2) | 0.027 |
| IL.23, Median (IQR) | 14.8 (5.3, 42.9) | 21.0 (8.1, 43.7) | 14.1 (5.3, 22.5) | 11.7 (4.6, 36.1) | 31.5 (12.6, 48.8) | 0.078 |
| IL.33, Median (IQR) | 31.5 (6.0, 69.2) | 39.2 (15.1, 79.1) | 41.3 (18.2, 91.0) | 28.1 (5.5, 61.1) | 30.4 (1.8, 68.5) | 0.592 |

Note: Data are presented as median (IQR). Cytokine concentrations are measured in pg/mL. The p values were calculated using Kruskal-Wallis test for comparisons across the four groups defined by CD4 count threshold of 50 cells/μL and TYG index threshold of 9.22.

****Abbreviations:**** CD4, CD4+ T-cell count; TYG, triglyceride-glucose index; IQR, interquartile range; IL, interleukin; IFN, interferon; TNF, tumor necrosis factor; MCP, monocyte chemoattractant protein.

**Supplementary Table 8.Plasma Cytokine Levels in Severely Immunosuppressed (CD4<50 cells/μL) HIV Patients with Sepsis: Low TyG vs. High TyG**

| **Variables** | **Total (n = 117)** | **​​CD4<50 & TYG<9.22 (n = 95)** | **​​CD4<50 & TYG≥9.22 (n = 22)​** | **p** |
| --- | --- | --- | --- | --- |
| IL.1b, Median (IQR) | 1.3 (0.0, 4.9) | 1.3 (0.0, 4.8) | 1.4 (0.0, 9.8) | 0.528 |
| IFN.a2, Median (IQR) | 7.5 (3.2, 16.1) | 7.2 (3.2, 13.8) | 10.1 (2.9, 20.3) | 0.78 |
| IFN.y, Median (IQR) | 13.9 (6.2, 28.6) | 14.0 (6.1, 27.9) | 13.1 (7.2, 27.7) | 0.778 |
| TNF.a, Median (IQR) | 5.4 (0.0, 17.3) | 5.2 (0.0, 14.5) | 5.4 (1.3, 26.3) | 0.284 |
| MCP.1, Median (IQR) | 448.5 (206.8, 864.3) | 419.6 (211.6, 867.5) | 452.3 (196.8, 682.9) | 0.884 |
| IL.6, Median (IQR) | 9.1 (3.5, 24.5) | 7.7 (3.4, 26.9) | 11.7 (4.4, 19.5) | 0.403 |
| IL.8, Median (IQR) | 9.9 (0.8, 39.6) | 9.2 (0.5, 41.3) | 12.6 (3.1, 32.0) | 0.676 |
| IL.10, Median (IQR) | 27.9 (15.3, 76.2) | 27.3 (15.1, 69.4) | 45.0 (18.4, 91.8) | 0.289 |
| IL.12p70, Median (IQR) | 1.6 (0.0, 5.1) | 1.6 (0.0, 4.9) | 0.9 (0.0, 5.3) | 0.69 |
| **IL.18, Median (IQR)** | **2133.5 (1222.5, 4983.0)** | **1991.3 (1166.0, 4357.5)** | **4811.6 (1938.0, 8332.2)** | **0.009** |
| **IL.23, Median (IQR)** | **14.7 (4.9, 43.0)** | **11.7 (4.6, 36.1)** | **31.5 (12.6, 48.8)** | **0.025** |
| IL.33, Median (IQR) | 28.1 (5.0, 61.1) | 28.1 (5.5, 61.1) | 30.4 (1.8, 68.5) | 0.922 |

Note: Data are presented as median (IQR). Cytokine concentrations are measured in pg/mL. The p values were calculated using Kruskal-Wallis test for comparisons across the four groups defined by CD4 count threshold of 50 cells/μL and TYG index threshold of 9.22.

****Abbreviations:**** CD4, CD4+ T-cell count; TYG, triglyceride-glucose index; IQR, interquartile range; IL, interleukin; IFN, interferon; TNF, tumor necrosis factor; MCP, monocyte chemoattractant protein.
